# Exploring the Role of the Private Sector in Tuberculosis Detection and Management in Lima, Peru: A Mixed-Methods Patient Pathway Analysis

**DOI:** 10.4269/ajtmh.23-0504

**Published:** 2024-05-14

**Authors:** Christoph Wippel, Sheyla Farroñay, Hannah N. Gilbert, Ana Karina Millones, Diana Acosta, Isabel Torres, Judith Jimenez, Valentina A. Alarcón, Leonid Lecca, Courtney M. Yuen

**Affiliations:** ^1^ Department of Global Health and Social Medicine, Harvard Medical School, Boston, Massachusetts;; ^2^ Socios En Salud Sucursal Peru, Lima, Peru;; ^3^ Dirección de Prevención y Control de la Tuberculosis, Ministry of Health, Lima, Peru;; ^4^ Division of Global Health Equity, Brigham and Women’s Hospital, Boston, Massachusetts

## Abstract

In Latin America, little is known about the involvement of private health-care providers in tuberculosis (TB) detection and management. We sought to gain a better understanding of current and potential roles of the private sector in delivering TB services in Peru. We conducted a mixed-methods study in North Lima, Peru. The quantitative component comprised a patient pathway analysis assessing the alignment of TB services with patient care-seeking behavior. The qualitative component comprised in-depth interviews with 18 private health-care providers and 5 key informants. We estimated that 77% of patients sought care initially at a facility with TB diagnostic capacity and 59% at a facility with TB treatment capacity. Among private facilities, 43% offered smear microscopy, 13% offered radiography, and none provided TB treatment. Among public-sector facilities, 100% offered smear microscopy, 26% offered radiography, and 99% provided TB treatment. Private providers believed they offered shorter wait times and a faster diagnosis, but they struggled with a lack of referral systems and communication with the public sector. Nonrecognition of private-sector tests by the public sector led to duplicate testing of referred patients. Although expressing willingness to collaborate with public-sector programs for diagnosis and referral, private providers had limited interest in treating TB. This study highlights the role of private providers in Peru as an entry point for TB care. Public–private collaboration is necessary to harness the potential of the private sector as an ally for early diagnosis.

## INTRODUCTION

Tuberculosis (TB) has once again surpassed COVID-19 to reclaim its position as the world’s deadliest infectious disease, responsible for approximately 1.6 million deaths in 2021.^1^ Nearly one third of all people with TB may not receive an accurate diagnosis and suitable care.^1^ Limited access to health-care services and insufficient means of detecting cases contribute to delayed or missed diagnoses.^2^ To ensure timely diagnosis for all patients, TB services must be accessible where people with TB seek care.

Patient pathway analyses (PPAs) from multiple countries revealed that a sizable portion (20–90%) of people with TB seek care first in the private sector.^3^^–^^7^ After this initial care seeking, many people with TB visit multiple facilities, moving between private and public sectors, before receiving a TB diagnosis.^8^^–^^11^ Despite the importance of the private sector in the pathway to diagnosis for many people with TB, many national TB programs engage less with private providers than the public health-care system. Some PPA studies have revealed a lack of basic information on TB diagnostic service availability in the private sector, as government regulatory agencies do not cover private facilities comprehensively.^3^^,^^7^^,^^12^ Yet in Asia and Africa, engaging the private sector has been shown consistently to increase TB case detection and improve treatment outcomes, and various models for partnership exist.^13^

Despite private health-care providers playing an increasingly significant role in health-care delivery in Latin American nations, the literature on the involvement of private health-care providers in TB detection and management for this region is limited. Studies from Mexico, Nicaragua, and Peru have shown that 22%, 35%, and 41% of patients, respectively, initially seek care in private-sector facilities.^11^^,^^14^^,^^15^ A study from Brazil^16^ found that 9% of TB diagnostic tests were performed in the private sector in 2012, representing an increase from a decade earlier. However, among the 78 studies included in a systematic review of public–private partnership models for TB,^13^ only one originated from Latin America, and it focused on the involvement of private pharmacies rather than private health facilities.^17^ We sought to gain a better understanding of current and potential roles of the private sector in TB diagnosis and management in Peru. We therefore carried out a mixed-methods study, combining the first TB PPA in a Latin American country with a qualitative examination of private-sector providers’ perspective on their role in TB care.

## MATERIALS AND METHODS

We used a convergent mixed-methods design. The quantitative component comprised a PPA in which we sought to understand TB diagnostic and treatment capacity in health facilities where people with TB first seek medical care.^18^ Qualitative data were collected to elaborate, clarify, and explain the quantitative results by exploring the role of private health-care providers in TB care. Although the PPA encompasses all sectors of the health-care system, the qualitative data focus specifically on the private sector because public-sector TB services are, in general, better understood.^19^^–^^21^

### Study setting.

Peru’s public health-care sector mainly comprises the Ministry of Health (MINSA) system, which serves people with government insurance provided to those who lack other forms of insurance, and the EsSalud system, which is affiliated with the Ministry of Labor, and which serves people with employer-funded social insurance.^22^ The private sector provides services to people who pay out of pocket or who possess private insurance, as well as patients from the EsSalud system who can receive services through affiliated private facilities. In the 2022 National Household Survey,^23^ of the Lima residents who reported seeking care at any health facility in the past 4 months, 61% used a MINSA or EsSalud facility and 39% used a private facility. The Ministry of Health establishes national health-care standards, whereas the independent National Health Regulatory and Supervisory Agency (SUSALUD) is responsible for ensuring that health-care facilities in all sectors comply with standards. By law, private-sector facilities are not allowed to purchase TB drugs.

Lima, the capital of Peru, is divided into four administrative regions. We aimed to conduct a PPA for the northern Lima region, which comprises nine districts and a population of ∼2.5 million. Although data on public health system services, case notifications, and treatment outcomes were available for North Lima, no data were available on TB services in the private sector, and we had to conduct surveys to obtain this information. For feasibility, we limited the surveys to Carabayllo, one district of North Lima, and extrapolated the results to the other districts. At the time of the study, Carabayllo had an estimated 13% of the population of North Lima with a slightly lower TB notification rate than North Lima as a whole (96 versus 110 per 100,000 population) and a similar percentage of TB cases reported from the MINSA system (89% for Carabayllo versus 83% for North Lima). Private facilities of all types, from small clinics to hospitals, are present in Carabayllo, and in the 2022 National Household Survey,^23^ private-sector use among people who used a health facility in the past 4 months was similar to the region (36% for Carabayllo versus 33% for North Lima).

### Patient pathway analysis.

Our study’s quantitative component comprised a PPA, a standardized approach to evaluate the alignment between care-seeking behavior and TB services.^18^ Data on initial location of health care–seeking among people with TB was drawn from a 2017 study^11^ from Lima; these values were consistent with patient surveys we conducted during 2020 through 2022 in North Lima (unpublished data). We obtained data on TB service availability (smear microscopy, chest radiography, and treatment) in the private sector via brief surveys, conducted in 2022, with providers working in Carabayllo private health-care facilities. Also in 2022, we obtained data on these services in North Lima MINSA facilities from the North Lima regional health authority and in the EsSalud network from their public websites. We used WHO^1^ TB incidence estimates for 2021 to estimate overall case detection because no subnational estimates were available. From the national TB program, we obtained data on notifications and treatment outcomes for North Lima patients diagnosed in 2021.

To estimate the number of private-sector facilities where a person with TB could seek care, we acquired a list of all registered private health-care facilities in North Lima from SUSALUD in May 2022; facilities are required by law to register with SUSALUD to operate. We filtered the list to categories that included general medical attention, then excluded those that provided only specialist care not relevant to TB (e.g., dental, psychological, ophthalmological). We visited the remaining registered facilities in Carabayllo and determined how many were functioning, because we realized that facilities were not necessarily removed from the list if they were no longer functioning. We used the ratio of functioning facilities to registered facilities to estimate the total number of functioning private facilities in North Lima.

We categorized MINSA facilities into primary care centers and hospitals. We considered EsSalud and private-sector facilities each as a single category because distinctions between different types of facilities were not standardized. We calculated diagnostic coverage of smear microscopy and radiography, defined as the proportions of facilities with these capabilities. Rapid molecular testing is not widely performed as a first-line diagnostic test in Peru. We estimated the percentage of people with TB who had diagnostic access at initial care-seeking (defined as first seeking care at a facility offering either smear microscopy or radiography) by multiplying the proportion of people with TB who first sought care at a particular type of facility in the 2017 study^11^ with the calculated diagnostic coverage for that type of facility. We used a similar approach to estimate treatment coverage (the proportion of health facilities that provided TB treatment) and treatment access at initial care-seeking (the proportion of people who first sought care at a facility offering TB treatment). We estimated notification location (the proportion of people with TB who were diagnosed and notified, by sector) by combining North Lima TB notification data with the WHO’s^1^ 2021 estimate of Peru’s TB case detection rate, because subnational estimates of case detection rate were not available. We report treatment outcomes for the 2021 patient cohort from North Lima, combining all sectors.

### Qualitative data collection and analysis.

We recruited for interviews key informants who were knowledgeable about policies that affect TB services in the private sector and health-care providers working in the private sector in North Lima. We used existing professional connections of the study team to recruit key informants, which included representatives of the North Lima regional health authority, national-level MINSA, SUSALUD, and Socios En Salud, a nonprofit nongovernmental organization (NGO) active in the field of TB care. We recruited private-sector providers from among those who completed the PPA survey. We sampled purposively an equal number of participants who reported having diagnosed TB within the past year and those who had not (which was recorded in the survey). Because our PPA survey sample had a limited number of providers who had diagnosed TB, we used snowball sampling to reach our initial target for interviews, requesting interviewees to refer other information-rich private health providers practicing in North Lima for participation. In total, we conducted 22 interviews involving 23 participants: 5 key informants, 12 private providers who were a subset of the survey sample, and 6 additional private providers identified through snowball sampling. Eight interview participants were female and 15 were male. All but two of the private providers were medical doctors (one was a nurse and one was a midwife). Ten survey participants were not interested in being recruited for interviews, and no recruited interview participants declined.

We conducted one-time interviews using semistructured interview guides (Supplemental Appendix 1). Interviews were conducted in Spanish by trained Peruvian nurse technicians (S. Farroñay, D. Acosta, and I. Torres) working for the NGO Socios En Salud, all of whom had experience in qualitative research. The interviewers did not know the participants previously, except for one key informant who worked for Socios En Salud in a different department. The interviewers’ training and experience afforded them familiarity with TB and the local treatment landscape, which they leveraged to formulate clarifying follow-up questions and relevant probes to elicit in-depth data from the participants. Interviews lasted 30–90 minutes. Three interviews were conducted via videoconference and the rest were held in person at participants’ workplaces. All interviews were audio recorded, and a note-taker was present during each interview (either one of the other interviewers or C. Wippel, a medical doctor).

Interview transcripts were created using artificial intelligence–assisted software (HappyScribe, Barcelona, Spain). Transcripts were checked and corrected by S. Farronay and C. Wippel. C. Wippel translated the transcripts into English. S. Farronay provided knowledge about the local context to ensure accurate translation. C. Wippel open-coded a subset of high quality transcripts, discussed the results with H. N. Gilbert (a medical anthropologist) and C. M. Yuen (an epidemiologist), and developed a final codebook with 34 codes. C. Wippel coded the entire data set using Dedoose v. 9.0.17 (SocioCultural Research Consultants LLC, Los Angeles, CA). C. Wippel, H. N. Gilbert, and C. M. Yuen analyzed the coded data using an inductive approach, generating a preliminary set of descriptive categories that were refined through iterative examination. L. Lecca helped with interpretation. The analysts’ different backgrounds supported the rigor of the analytic process. C. Wippel’s presence during data collection ensured accurate interpretation. L. Lecca’s 18 years of experience working at a Peruvian NGO that supports TB patients allowed contextualization of findings within the Peruvian health-care landscape. C. M. Yuen’s 11 years of experience studying TB services in multiple settings, including Peru, allowed broader contextualization. H. N. Gilbert’s role as a qualitative researcher working outside of Peru provided an independent check throughout the analytic process.

We used a content analysis approach to data analysis.^24^ We integrated quantitative and qualitative results by overlaying the qualitative insights onto the PPA indicators using a joint display technique.^25^ This approach allowed us to understand how the private sector might serve as a facilitator or barrier to TB care at different points along the patient pathway.

## RESULTS

**Figure 1.**
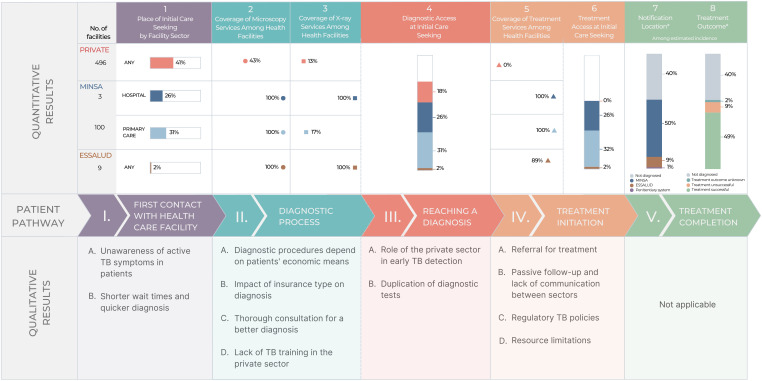
Joint display of integrated results presented longitudinally across the patient pathway. Quantitative results are stratified by sector, considering the private sector, the public system operated by the Ministry of Health (MINSA), and the public system operated by the Ministry of Labor (ESSALUD). * Notification data were used from 2021, when case notifications had decreased by approximately 20% during the COVID-19 pandemic; therefore, the percentage of missed people with tuberculosis (TB) is greater than in prior years.

Figure 1 shows the quantitative PPA results and qualitative themes structured around the main components of the patient pathway. Tables 1 to 3 show supporting quotes for qualitative themes corresponding to components I through IV of the patient pathway illustrated in Figure 1.

**Table 1:** Quotes illustrating themes related to first contact with health facilities (step I in the patient pathway)

Theme	Quote
I-A. Unawareness of active TB symptoms in patients	*“I think that the main reason why a person would come to a private establishment with tuberculosis is to learn about their disease. They might confuse the symptoms and think that it is another respiratory problem and not TB, because most people know that TB is managed in public centers.”* (Interview 10, private provider who diagnosed TB in the past year)
I-B. Shorter wait times and quicker diagnosis	“*If there is suspicion [for TB], we request a sputum BK [smear microscopy] test. This BK test can be done privately or publicly at MINSA. Publicly, it’s free, but the results take a month. . . . The patient arrives at the public sector with a diagnosis, and what does the TB program do? They start the treatment. You skipped the whole month or 2 months that the pulmonologist would have taken to get the X-rays at MINSA, the CT scan, and the BK test. That time was shortened, and you already have the diagnosis and can start treatment.* (Interview 18, private provider who diagnosed TB in the past year)

BK = smear microscopy; CT = computed tomographic; MINSA = Ministry of Health; TB = tuberculosis.

**Table 2:** Quotes illustrating themes related to the diagnostic process and reaching a diagnosis (steps II and III in the patient pathway)

Theme	Quote
II-A. Diagnostic procedures depend on patients’ economic means	*“Normally in all private centers, we have more access to blood, ultrasound, and radiological tests, because the person who goes to a [private] clinic usually has the economic means to afford it. So, we reach a diagnosis more easily because we ask for everything that is needed, they do all the tests, and the diagnosis is made quickly.”* (Interview 17, private provider who diagnosed TB in past year)
	*“The cost of the consultation is low and they make the most money with the laboratory. For example, with [facility name], I know of many patients I have seen with TB that have gone through [facility name] and they all come with spirometry and many expensive tests as well. And they did not do a smear because it was very cheap.”* (Interview 1, key informant from NGO)
II-B. Impact of insurance type on diagnosis	*“If I were in a clinic that had everything, and the patient had an EPS [private insurance] that covers everything, I would order everything, from a sputum BK [smear microscopy] to a tomography scan. But we are talking about the poorest portion of the population. We have to think carefully about what we order for them because sometimes the patient does not have the means to pay for everything. . . . I take a good medical history and a physical exam, and if they have respiratory symptoms of a contagious disease such as tuberculosis, I order them to get a serial sputum BK, usually because those are the cheapest: the sputum BK and a chest X-ray.”* (Interview 10, private provider who diagnosed TB in past year)
II-C. Thorough consultation for a better diagnosis	*“Ideally, they [health-care providers] take their time to take a good medical history for the patient. If they achieve this, it’s already 80%; the diagnosis is 80% in the medical history. . . . They have to dig for things, and then they will find out what the problem is.”* (Interview 2, private provider who diagnosed TB in past year)
II-D. Lack of TB training in the private sector	*“Here, in this establishment, I have not received any training, neither for diagnosis nor for management of TB. I have been practicing here in the country since [date] 2020. . . . Until now, I have not had any training here, at least not with respect to TB.”* (Interview 5, private provider who did not diagnose TB in past year)
III-A. Role of private sector in early TB detection	*“Well, in our case as a country, as I mentioned, one of the main advantages that could be considered for their case is early detection—that is, the ability to detect or at least identify probable cases for quick referrals. I mean, in that they could contribute a lot.”* (Interview 22, key informant from MINSA)
III-B. Duplication of diagnostic tests	*“There may be requests for sputum smear tests to private laboratories, but we do not know if they are truly capable of providing that type of test. . . . It has always been considered that if diagnoses are made in the private sector, they must be validated because if they are not evaluated by the National Health Institute that gives a certain guarantee, we will not have the confidence in knowing that those diagnoses are truly what they are.”* (Interview 22, key informant from MINSA)

BK = smear microscopy; EPS = a type of private insurance; MINSA = Ministry of Health; NGO = nongovernmental organization; TB = tuberculosis.

**Table 3:** Quotes illustrating themes related to treatment initiation (step IV in the patient pathway)

Theme	Quote
IV-A. Referral for treatment	*“Unfortunately, the private sector refers patients to the MINSA system for treatment because they don’t have the treatment regimens. It would be ideal to give the patient a week of treatment and instruct them to follow up at a nearby health center for monitoring, but that’s not something that we are currently doing.”* (Interview 20, private provider who diagnosed TB in the past year)
IV-B. Passive follow-up and lack of communication between sectors	*“Patients usually reach out to me . . . and update me on the progress of the tests and results or if they have been cleared. So it is the opposite. Patients reach out to me rather than me reaching out to them.”* (Interview 13, private provider who did not diagnose TB in the past year)
IV-C. Regulatory TB policies	*“I am not familiar with any regulations in the private sector for TB. What I do know is that all TB cases should be referred to the nearest MINSA facility.”* (Interview 18, private provider who diagnosed TB in the past year)
	*“There are private facilities that, depending on the level of complexity, are definitely capable of treating tuberculosis . . . . They should treat tuberculosis. They are prepared. They even have their pulmonologist, and they have their areas. That is why this should be only reserved for the [more complex] clinics, because now they have to comply with new regulations.”* (Interview 3, private provider who did not diagnose TB in the past year)
IV-D. Resource limitations	*“And seeing it from the point of a private provider, I think it would be a bit difficult, financially speaking [to have a pulmonologist on standby in case we need one]. So, I am seeing it from the perspective of how much that would cost me.”* (Interview 11, private provider who did not diagnose TB in the past year)

MINSA = Ministry of Health; TB = tuberculosis.

### First contact with health-care facility.

In North Lima in 2022, there were 1,687 registered private health facilities, of which we estimated that 859 provided general medical services. In the district of Carabayllo, we were able to find 26 functioning private facilities compared with the 45 registered. Applying a similar ratio throughout North Lima yielded an estimated 496 functioning private facilities. Public health facilities in North Lima with general medical services included 100 MINSA primary care centers, 3 MINSA hospitals, and 9 EsSalud facilities. A previous study from Lima^11^ found that 41% of people with TB first sought care in the private sector, 31% at MINSA primary care centers, 26% at MINSA hospitals, and 2% at EsSalud facilities.

#### Lack of awareness of TB symptoms.

Private health-care providers perceived that although the general population is aware that treatment of TB is provided in public programs, people may not recognize their symptoms as indicative of TB and therefore initially seek care in a private facility, which may be the nearest or most convenient facility.

#### Shorter wait times and quicker diagnosis.

Private health-care providers viewed shorter wait times for appointments and faster diagnostic results as reasons why patients seek care in the private sector. By providing patients with earlier appointments, faster test results, and more thorough consultations, private providers believed they could make a TB diagnosis sooner and thereby reduce delays to treatment initiation compared with the public sector.

### Diagnostic process.

Providers from 23 of the 26 (88%) private facilities in Carabayllo agreed to take the survey on TB service availability. Smear microscopy was offered in 10 (43%) surveyed private facilities, 100 (100%) MINSA primary care centers, 3 MINSA (100%) hospitals, and 9 (100%) EsSalud facilities. Radiography was offered at 3 (13%) surveyed private facilities, 17 (17%) MINSA primary care centers, 3 (100%) MINSA hospitals, and 9 (100%) EsSalud facilities.

#### Diagnostic procedures depend on patients’ economic means.

Private providers believed that one of the strengths of the private sector is the ability to arrive at a faster diagnosis in part because of the availability of more advanced technologies. They argued that collaborations with private laboratories and more advanced diagnostic technologies such as magnetic resonance imaging, computed tomographic scans, and GeneXpert in better equipped facilities can promote faster diagnosis. However, the accessibility of these services is dependent on the patient’s financial means, as they typically pay out of pocket. A key informant from an NGO mentioned that certain private facilities may bypass basic tests such as smear microscopy and proceed immediately with more advanced, and sometimes unnecessary, tests.

#### Impact of insurance type on diagnosis.

Although some private providers indicated that patients’ insurance status did not affect the quality or type of services they received, because services were all paid for out of pocket, others noted significant disparities in diagnostic services depending on insurance coverage and the patient’s economic means. These providers explained that they tended to offer patients who had private insurance more advanced and expensive tests, whereas those without insurance or with state insurance were more likely to be offered basic and cheaper tests.

#### Thorough consultation for a better diagnosis.

The majority of private providers interviewed exhibited familiarity with the clinical indicators that raise suspicion of active TB. They noted that combining these with a thorough social history, including details such as socioeconomic status, living conditions, and social contacts, aids the diagnostic process considerably.

#### Lack of TB training in the private sector.

However, private health-care facilities do not provide any training for their staff on TB, and there are no external training programs available for private providers. As a result, private providers’ knowledge of TB is based largely on their prior medical training in academic institutions or their experience working in the public sector. Some private providers reported engaging in self-training out of personal interest.

### Reaching a diagnosis.

Overall, we estimate that 77% of people with TB initially sought care at a facility with a TB diagnostic capacity (sputum smear microscopy or radiography). The lack of TB services at the initial care-seeking location was driven by the people who initially went to a private facility, because all public health facilities offered at least smear microscopy. Of the surveyed private providers, eight (35%) reported they had diagnosed at least one person with TB in the past year.

#### Role of the private sector in early TB detection.

Private providers agreed that the private sector’s main role lies in the early detection and diagnosis of TB as opposed to treatment. They perceived the availability of resources, speed of diagnosis, and access to cutting-edge technologies as strengths of the private sector. A key informant from MINSA agreed that there was value in the private sector’s contribution to early TB detection, and expressed a general openness to expand the role of the private sector in this capacity. Some providers proposed expanding the private sector’s early detection potential with more widespread use of advanced diagnostic tests, such as GeneXpert, or by implementing a screening program that would test every patient with respiratory symptoms for TB.

#### Duplication of diagnostic tests.

Some private providers described having diagnosed patients with TB and referred them to the public sector, only to have the diagnostic test results rejected by the public sector and repeated, causing distress to the patient. The Ministry of Health’s key informant explained that this stems from a lack of oversight and validation of private laboratories, which are not supervised or monitored as part of the public network, leading to lower levels of confidence in their results.

### Treatment initiation.

None of the private providers offered treatment of TB, whereas treatment was available at all MINSA facilities and eight (89%) of the EsSalud facilities. Thus, we estimate that 59% of people with TB had access to treatment services at their initial care location.

#### Referral for treatment.

There was a clear consensus that the private sector does not provide TB treatment, with interviewees citing legal prohibitions, medication inaccessibility, and the belief that the public program is performing well. Although many private providers asserted that the private sector’s responsibility ends with referral of people with TB to the public sector, one suggested they should have the option of providing initial treatment after diagnosis until the patient reaches the public sector to avoid delay in starting treatment.

#### Passive follow-up and lack of communication between sectors.

Upon referral to the public sector, the responsibility for treatment often shifts to patients themselves. For most private providers, the referral process is viewed as a handover to the public program, with the expectation that the patient will receive appropriate treatment. There are no standardized systems for sharing patient information from the private sector to the public sector after a patient is diagnosed with TB and referred, or to inform the referring private facility that a patient has arrived and initiated treatment in the public facility. Private providers expressed the desire for better feedback from the MINSA system about the status of referred patients. A digital platform for sharing patient information exists for public facilities, but private facilities are currently excluded. During the COVID-19 pandemic, this system was made accessible to private facilities for the purposes of sharing vaccine-related data with the public sector, which raised the question of why something similar could not be achieved for TB.

#### Regulatory TB policies.

Private health-care providers believed that regulatory policies prohibit the private sector from treating TB patients, although they did not generally know the details of the policies. However, the NGO key informant mentioned that regulatory policies provide for a mechanism that could allow private facilities to enter into an agreement with MINSA to treat drug-sensitive TB. However, the private facility would have to fulfill strict requirements at their own cost, including specialized staff, provisions for contact tracing, follow-ups, supplemental services, and biosafety. No private facility in Peru had entered into such an agreement at the time of our study. High levels of bureaucracy and few financial incentives were mentioned as possible reasons for the low levels of interest in this mechanism.

#### Resource limitations.

Many private providers expressed concerns about the feasibility of delivering quality TB care in their facilities, considering the financial implications of the diverse services required to address patients’ needs. Specialist staff such as pulmonologists, nutritionists, and psychologists are often not part of the regular staff of private facilities, and it can be challenging financially to retain them on standby. In addition, the lack of adequate space and a biosafety infrastructure was mentioned as a possible limitation for the provision of treatment in the private sector.

### Treatment outcome.

The WHO estimated that 40% of incident TB cases were reported in Peru in 2021.^1^ Among all TB cases reported in North Lima in 2021, 83% were reported by MINSA facilities, 15% by EsSalud facilities, and 2% by the penitentiary system. No cases were reported by the private sector. Among people who initiated treatment in North Lima, 82% were treated successfully and 8% were lost to follow-up. Thus, an estimated 49% of all people with TB were treated successfully, given that 40% were not diagnosed.

## DISCUSSION

Our study highlights the role of the private sector in the diagnosis of TB in North Lima, Peru, and the need for enhanced public–private collaboration. Private facilities greatly outnumber public facilities in this setting, and many people with TB use the private sector despite having to pay out of pocket.^11^ However, the limited availability of TB diagnostic services in the private sector means that approximately one quarter of people with TB are estimated to have initially sought care at locations without TB diagnostic services. The private providers we interviewed placed the responsibility for treatment on the public sector, but struggled with a lack of standardized referral systems and poor communication with the public sector. In addition, nonrecognition of tests performed in the private sector leads to duplication of tests and delayed treatment initiation, which could potentially prolong infectiousness.

Our findings highlight the potential benefits of closer collaboration between private providers and the Peruvian national TB program to improve TB detection. Although studies from other settings have generally documented the willingness of private providers to engage with the national TB program,^26^ some studies have documented low motivation and even resistance to transferring patients to public-sector facilities.^27^^,^^28^ Providers in our study were comfortable with referring TB patients to the public sector for treatment and did not make a clear call for treatment in the private sector. The stringent regulatory environment, the goal of which is to prevent substandard TB treatment in the private sector and thus the amplification of drug resistance, appears to have created a financial disincentive for private-sector treatment in Peru.

Although our qualitative findings depicted a generally positive outlook concerning TB diagnosis in the private sector, with private providers believing they were performing well, our quantitative results indicate that most private facilities lacked TB diagnostic capabilities, and a minority of surveyed private providers had diagnosed a case of TB in the past year. In part, this inconsistency is likely attributable to the characteristics of our interview sample. Providers who agreed to interviews were predominantly from facilities with diagnostic capacities, and we sampled providers purposively such that half had diagnosed TB recently. Even so, the interviewed providers who had not recently diagnosed TB did not highlight challenges or limitations to TB diagnosis in the private sector. This suggests either that they were unaware of these limitations or that social desirability bias prevented them from discussing these issues. Studies^29^ of the quality of private-sector TB diagnostic services in Asian and African countries have found wide variation in quality within settings, and have documented low TB testing rates, even for people with typical TB symptoms. It is likely that such variation in quality exists in Peru and other Latin American countries as well, and that diagnoses are being missed in the private sector.

The main barrier that hinders effective cooperation between the private and public sectors in Peru is the lack of communication and information sharing. Effective strategies for public–private partnership include the national TB program providing technical support to private providers and establishing formal collaborative relationships with defined expectations.^13^ In Peru, MINSA could help harness the potential of the private sector by providing training for private providers and by facilitating private facilities to register as certified diagnostic support centers so that public facilities can accept their diagnostic test results without repeating them. Successful public–private partnerships have also highlighted the importance of formal feedback mechanisms that inform private providers about what happens to their patients who have been referred to the public sector for treatment.^30^^,^^31^ In Peru, establishing formal referral mechanisms through standardized paper forms and access to the electronic surveillance system could facilitate prompt treatment. This could potentially enable private-sector providers to initiate treatment before referral with drugs obtained from MINSA. Without an effective referral system, private-sector treatment initiation could risk loss to follow-up. Moreover, direct private-sector reporting of TB diagnoses through the national electronic surveillance system could improve case detection, as has been observed in India.^32^

One strength of our study is the use of in-depth interviews with private health-care providers in combination with a PPA. This approach provides insight into what happens when people with TB seek care in the private sector, which was poorly understood in our setting, as in other Latin American countries. There are several limitations to our research. First, because the list of private facilities was not updated to reflect closures and data were not available on TB services in the private sector, we collected data by directly identifying facilities and conducting surveys of TB service availability. This was only feasible to do for a single district. Carabayllo is one of the larger but less urbanized districts of North Lima, so extrapolating the findings from this district to others may underestimate service availability in the private sector in North Lima. In addition, we relied on health-care–seeking data from a prior study of people with TB in Lima,^11^ which included only people being treated within the MINSA system. People treated in the EsSalud system are more likely to have used EsSalud facilities as well as private facilities as a result of existing insurance partnerships. Given that EsSalud facilities have TB services and private facilities may or may not, it is unclear how including these individuals would change the results of the PPA, although the magnitude of any changes would likely be small, because only 15% of people with TB are treated in the EsSalud system. Last, the qualitative portion of our study relied in part on snowball sampling, which was instrumental in identifying knowledge-rich participants to complete our qualitative sample, but which is subject to selection bias.

## CONCLUSION

In conclusion, our study highlights the role private providers play as an entry point of care for a sizable portion of people with TB, even in a country where treatment is restricted to the public sector. Strengthening linkages between the private and public sectors by establishing a formal referral system and improving communication between sectors could lead to higher case detection rates and better continuity and timeliness of care for people with TB, both of which could contribute to reducing transmission. To harness the potential of the private sector in TB diagnosis in Peru, it is necessary to begin a dialogue between the public and private sectors to explore possible public–private partnership models.

## Supplemental Materials

10.4269/ajtmh.23-0504 Supplemental Materials
